# Portal vein tumour thrombosis radiotherapy improves the treatment outcomes of immunotherapy plus bevacizumab in hepatocellular carcinoma: a multicentre real-world analysis with propensity score matching

**DOI:** 10.3389/fimmu.2023.1254158

**Published:** 2023-10-19

**Authors:** Cuiping Tang, Qin He, Jian Feng, Ziyue Liao, Yunli Peng, Jian Gao

**Affiliations:** ^1^ Department of Gastroenterology and Hepatology, Second Affiliated Hospital of Chongqing Medical University, Chongqing, China; ^2^ Department of Graduate, The Second Clinical College of Chongqing Medical University, Chongqing, China; ^3^ Department of Gastroenterology and Hepatology, The First People's Hospital of Mianyang (SiChuan Mianyang 404 Hospital), Sichuan, China; ^4^ Department of Oncology, Bishan Hospital of Chongqing Medical University, Chongqing, China; ^5^ Department of Gastroenterology and Hepatology, Bishan Hospital of Chongqing Medical University, Chongqing, China

**Keywords:** hepatocellular carcinoma, portal vein tumour thrombosis, radiotherapy, immune checkpoint inhibitors, bevacizumab

## Abstract

**Background:**

This study aimed to evaluate the efficacy and safety of sequential immune checkpoint inhibitors (ICIs) plus bevacizumab therapy after radiotherapy for portal vein tumour thrombosis (PVTT) in patients with hepatocellular carcinoma (HCC).

**Methods:**

Retrospective data were collected from 113 patients with HCC with PVTT. Patients in the PVTT radiotherapy (radiotherapy + ICIs + bevacizumab) and control groups (ICIs + bevacizumab) were enrolled according to propensity score matching (PSM) analysis (1:1). The differences in progression-free survival (PFS), objective response rate (ORR), disease control rate (DCR), and potential factors affecting PFS between the groups were analysed. The adverse events (AEs) were compared between the two groups.

**Results:**

There were 47 patients in the two groups after PSM (1:1). The differences in neutrophil and lymphocyte counts, neutrophil-to-lymphocyte ratio (NLR), CRP, and CD4, CD8, and CD4-to-CD8 ratio before and after radiotherapy for PVTT (*P* < 0.05) in the PVTT radiotherapy group were significant. The patients in the PVTT radiotherapy group had a longer PFS (median, 9.6 *vs.* 5.4 months, *P* < 0.001), and the PFS rates of 3, 6, 9, and 12 months were 97.87% *vs.* 94.19%, 80.85% *vs.* 44.68%, 53.19% *vs.* 6.38%, and 23.40% *vs.* 0.00%, respectively (*P* < 0.001). There were also significant differences in the ORR (48.94% *vs.* 27.66%, *P* = 0.0339) and DCR (97.87% *vs.* 82.98%, *P* = 0.0141) between the two groups, and no serious AEs were observed. Multivariate Cox analysis showed that AFP expression, gross classification of HCC, PVTT type, extrahepatic metastasis, PVTT radiotherapy, and reduction in PVTT were independent factors influencing PFS (*P* < 0.05).

**Conclusions:**

Sequential ICIs plus bevacizumab therapy after radiotherapy for PVTT in patients with HCC is safe and feasible and may further prolong the PFS of patients.

## 1 Introduction

Hepatocellular carcinoma (HCC) has the clinical characteristics of insidious onset, rapid progression, early recurrence, poor prognosis, and high morbidity and mortality (1). Approximately three in four liver blood samples come from the portal vein system, and HCC is prone to invade the portal vein system to form portal vein tumour thrombosis (PVTT), with an incidence of ~44–66.2% (2). Patients with HCC and PVTT often had liver reserve damage, tumour invasion, and portal hypertension manifestations. PVTT is one of the most severe prognostic factors of HCC, and the median survival time of the patients without treatment was 2.7–4.0 months (2). Percutaneous portal vein stenting can open the portal vein to protect liver function and reduce portal hypertension; however, it cannot prevent the progression of PVTT. However, there is no international consensus on the diagnostic and treatment criteria for PVTT complicated by HCC, which causes great difficulties in the selection of treatment and prediction of efficacy. Transhepatic arterial chemotherapy and embolisation (TACE) is the standard treatment for patients with unresectable HCC. However, TACE has lower efficacy and safety than hepatectomy for patients with HCC and PVTT (3). The current Barcelona clinic liver cancer (BCLC) classification of HCC with PVTT is at an advanced stage; therefore, sorafenib or lenvatinib is generally recommended as first-line therapy for these patients (2). Although evidence for the efficacy of systemic therapy for advanced HCC is expanding, data on treatment guidance for a subgroup of patients with HCC with PVTT remain limited.

The liver is the “immune preferential organ”, the immune system in the liver is not sensitive to foreign bodies for its functional needs, resulting in the escape of primary liver tumour cells from the immune system’s surveillance and attack, also known as “immune escape”. Immunocheckpoint inhibitors (ICIs) such as programmed death-1/programmed death ligand 1 (PD-1/PD-L1) enable autoimmune cells to play an anti-tumour role by relieving the inhibition of immune cells. The FDA approved nivolumab as a second-line treatment for patients with HCC after sorafenib treatment in 2017, marking the official entry into the immunological era of HCC treatment. With the release of clinical results, CheckMate040 (4), KEYNOTE-240 (5), KEYNOTE-224 (6), SHR-1210 (7), pembrolizumab (PD-1), and atezolizumab (PD-L1) have been recommended as treatment options for HCC in multiple clinical guidelines, both domestically and overseas. Clinical research IMbrave 150 (8) and ORIENT-32 (9) showed better progression-free survival (PFS) and overall survival (OS) when ICIs plus bevacizumab were used as the first-line treatment for patients with advanced-stage HCC. However, the results of current clinical trials showed that the objective response rate (ORR) of ICIs plus bevacizumab treatment was still low. Therefore, there is an urgent need to explore combination treatments to improve treatment response rates.

The release of tumour antigens is an initial factor in the seven key links of immunotherapy. Tumour cells become necrotic after radiotherapy, and the immune system is fully activated, releasing tumour antigens (10). Therefore, PVTT radiotherapy combined with ICIs is theoretically feasible for the treatment of HCC. However, no relevant clinical studies have been conducted thus far. Our study aimed to investigate the changes in immune-related indicators after radiotherapy for PVTT in patients with HCC, to evaluate the efficacy and safety of sequential ICIs plus bevacizumab therapy after radiotherapy for PVTT, and to preliminarily explore the factors affecting the efficacy in these patients.

## 2 Methods

### 2.1 Inclusion and exclusion criteria

Inclusion criteria: HCC was diagnosed clinically or pathologically according to the diagnostic criteria of the American Liver Association. All patients were found with PVTT by ultrasound B, computerized tomography (CT), magnetic resonance imaging (MRI) or digital subtraction angiography (DSA). Patients had no history of anti-tumour therapy and met the indications of medical and local treatments. None of them had received chemotherapy, targeted molecular drugs, PD-1/PD-L1 immunotherpay, et al. The control group was treated with ICIs plus bevacizumab as the first-line treatment, and the PVTT radiotherapy group was treated with radiotherapy of PVTT followed by ICIs plus bevacizumab. The interval between radiotherapy of PVTT and systemic treatment must less than 1 month; Eastern Cooperative Oncology Group performance status score (ECOG-PS score) 0-1; Child-Pugh class A or B; Complete follow-up data were available.

Exclusion criteria: Suspected non-PVTT formation, PVTT intervention and other treatment history, combined with severe heart, liver and renal insufficiency, unable to complete treatment, bleeding tendency, significantly prolonged coagulation time, international normalized ratio (INR) >1.5, ECOG-PS score ≥2, Child-Pugh class C or D, systemic treatment after more than 1 month of PVTT radiotherapy, accompanied by other primary tumour or serious disease. Patients with red-color sign, severe esophagogastric fundus varices, history of hematemesis, aggressive tumour which had struck a major blood vessel were excluded. Rigor criteria including blinding, randomization of groups, and power analysis are not relevant to the study.

### 2.2 Clinical data

According to the inclusion and exclusion criteria, patients with PVTT diagnosed in the Second Affiliated Hospital of Chongqing Medical University, Sichuan Mianyang 404 Hospital and Bishan Hospital Affiliated to Chongqing Medical University from January 1, 2020 to June 31, 2022 were collected and selected. The sex, age, smoking history, alcohol consumption, diabetes, hypertension, cardiovascular disease, China liver cancer staging (CNCL), Child-Pugh class, ECOG-PS score Cause of hepatitis, liver cirrhosis, Quantity of hepatitis B virus deoxyribonucleic acid (HBV-DNA), serum alpha fetoprotein (AFP) expression, tumour gross classification of primary liver cancer (giant, massive, nodular, diffuse), Classification of PVTT, tumour metastasis and ICIs treatment of every patients were recorded.

### 2.3 Treatment

#### 2.3.1 Radiotherapy

Intensity-modulated radiation therapy (IMRT) was used as external radiotherapy for PVTT. The radiotherapy target volume was delineated by a radiologist under CT guidance with a total dose of 45 Gy (3 Gy ×15 fractions) for planning target volume (PTV), and radiotherapy was performed weekly from Monday to Friday.

#### 2.3.2 ICIs + bevacizumab therapy

PD-1 inhibitors Sintilimab (injection, 100 mg/bottle, Xinda Biopharmaceutical (Suzhou) Co. Ltd) 200 mg every 3 weeks, Camrelizumab (injection, 200 mg/bottle, Suzhou Shengdia Biomedicine Co. Ltd) 200 mg every 3 weeks, or PD-L1 inhibitor Atezolizumab (injection, 1200 mg/bottle, Roche Diagnostics GmbH) 1200 mg every 3 weeks, plus bevacizumab (injection, 100 mg/bottle, Roche Pharma (Switzerland) Ltd. or Qilu Pharmaceutical Co. Ltd) 15 mg/kg every 3 weeks therapy was continued within 1–2 weeks after the end of PVTT radiotherapy. The control group received PD-1/PD-L1 inhibitors plus bevacizumab as first-line treatment. PD-1/PD-L1 inhibitors and bevacizumab were administered every 21 d until discontinuation, delay in intolerable side effects, or serious treatment-related adverse events (AEs).

### 2.4 Observe indicators

PVTT radiotherapy group: Hematological indicators including Albumin, total bilirubin (TBIL), Alamine aminotransferase (ALT), Aspartate aminotransferase (AST), prothrombin time (PT), hemoglobin, neutrophils, lymphocytes, C-reactive protein (CRP) within 3 days before radiotherapy and before systemic treatment (or within 2 weeks after the end of radiotherapy), and neutrophils-to-lymphocytes ratio (NLR) and CD4-to-CD8 lymphocytes ratio were calculated.

Follow-up: All patients underwent liver-enhanced CT or MRI every 6-8 weeks during the treatment. All patients were evaluated according to RECIST1.1 criteria and divided into complete response (CR), partial response (PR), stable disease (SD), and progressive disease (PD). Survival analysis: Progression-free survival (PFS) was defined as the time from initial treatment to first tumour progression, death, or the end of follow-up. The concept of PFS in our study refers to disease progression regardless of local (liver), distant (metastasis), or NVPT progression. The ORR was defined as the proportion of patients whose tumour volume reduced to a prespecified value and maintained a minimum duration and was calculated as the sum of CR and PR (CR+PR), whereas the disease control rate (DCR) was defined as the proportion of patients whose tumours had shrunk or remained stable for a certain period of time, including CR, PR, and SD cases (CR+PR+SD). PFS and AEs were analysed in both groups.

### 2.5 Statistical analysis

Propensity score matching (PSM) analysis was used to minimise potential confounders and selection bias and to balance the patient baseline characteristics between groups. The propensity score was estimated for each patient using a multivariate logistic regression model, and 1:1 group matching was performed using the nearest-neighbour matching method without replacement. Variables including sex, age, smoking history, alcohol consumption, diabetes, hypertension, cardiovascular disease, CNCL staging, Child-Pugh class, ECOG performance status score, cause of hepatitis, liver cirrhosis, quantity of HBV DNA, AFP expression, gross classification of primary liver cancer, classification of PVTT, tumour metastasis, and ICIs were matched. A calliper width of 0.2 standard deviations was set to prevent poor matching.

The primary endpoints of this study were PFS, ORR, and DCR, and the secondary endpoints were adverse events. Descriptive statistical methods were used to summarise the baseline characteristics of the patients. SPSS version 26.0 (RRID: SCR_002865, IBM, Armonk, New York, USA) (https://www.ibm.com/spss), and GraphPad Prism (version 9.0; RRID: SCR_002798, GraphPad Software, CA) (https://www.graphpad.com) were used to analyse the data. Statistical Tests and measurement data were analysed using t-tests. Enumeration data were analysed by χ^2^ test, Cox regression model was used for survival analysis, and *P* < 0.05 was considered statistically significant.

## 3 Results

### 3.1 General information

A total of 113 patients with complete data were screened according to the inclusion and exclusion criteria, of whom 55 were treated with ICIs plus bevacizumab after PVTT radiotherapy (PVTT radiotherapy group) and 58 were treated with ICIs plus bevacizumab therapy (control group). Overall, 47 patients in the PVTT radiotherapy group and 47 in the control group were enrolled in the PSM analysis (1:1), whereas eight patients in the PVTT radiotherapy group and 9 in the control group (17 patients) were excluded by PSM.

Characteristics including sex, age, smoking history, smoking history, drinking, diabetes, hypertension, cardiovascular disease, CNCL staging, Child–Pugh class, ECOG performance status score, cause of hepatitis, liver cirrhosis, quantity of HBV DNA, AFP expression, gross classification of primary liver cancer, classification of PVTT, tumour metastasis, and ICIs were matched and are shown in **Table 1**. There were no significant differences in the baseline characteristics between the two groups (*P* > 0.05).

**Table 1:** Characteristics of all patients in the two groups.

	Total group (n=113)		*P* value	PSM group (1:1) (n=96)		*P value*
	PVTT radiotherapy group(n=55)	Control group(n=58)		PVTT radiotherapy group (n=47)	Control group (n=47)	
**Sex**			0.91			0.77
Male	47 (85.45)	50 (86.21)		41 (87.23)	40 (85.11)	
Female	8 (14.55)	8 (13.79)		6 (12.77)	7 (14.89)	
**Age(years)**			–			–
Median	52	54		50	56	
Range	32-72	16-79		32-70	16-79	
**Smoking history**			0.92			0.53
Former	26 (47.27)	28 (48.28)		23 (48.94)	20 (42.55)	
Never	29 (52.73)	30 (51.72)		24 (51.06)	27 (57.45)	
**Alcohol consumption**			0.85			0.51
Yes	17 (30.91)	17 (29.31)		17 (36.17)	14 (29.79)	
No	38 (69.09)	41 (70.69)		30 (63.83)	33 (70.21)	
**Diabetes**			0.50			> 0.99
Yes	8 (14.55)	6 (10.34)		6 (12.77)	6 (12.77)	
No	47 (85.45)	52 (89.66)		41 (87.23)	41 (87.23)	
**Hypertension**			0.92			0.46
Yes	7 (12.73)	7 (12.07)		3 (6.38)	5 (10.64)	
No	48 (87.27)	51 (87.93)		44 (93.62)	42 (89.36)	
**Cardiovascular disease**			0.28			> 0.99
Yes	3 (5.45)	1 (1.72)		1 (2.13)	1 (2.13)	
No	52 (94.55)	57 (98.28)		46 (97.87)	46 (97.87)	
**CNCLstaging**			0.32			0.67
IIIa stage	34 (61.82)	41 (70.69)		28 (59.57)	30 (63.83)	
IIIb stage	21 (38.18)	17 (29.31)		19 (40.43)	17 (36.17)	
**Child-Pugh class**			0.30			0.37
A(5-6 score)	50 (90.91)	49 (84.48)		42 (89.36)	39 (82.98)	
B(7-9 score)	5 (9.09)	9 (15.52)		5 (10.63)	8 (17.02)	
**ECOG performance status score**			0.55			0.65
0	18 (32.73)	16 (27.59)		15 (31.91)	13 (27.66)	
1	37 (67.27)	42 (72.41)		32 (68.09)	34 (72.34)	
**Cause of hepatitis**			0.74			0.22
Hepatitis B(HBeAg/Carrier)	4 9(23/26) (89.09)	49 (15/34) (84.48)		43 (22/21) (91.49)	39 (12/27) (82.98)	
Hepatitis C	1 (1.82)	1 (1.83)		0 (0.00)	0 (0.00)	
NAFLD	5 (9.09)	8 (13.79)		4 (8.51)	8 (17.02)	
**Liver cirrhosis**			0.63			> 0.99
Yes	30 (54.55)	29 (50.00)		26 (55.32)	26 (55.32)	
No	25 (45.45)	29 (50.00)		21 (44.68)	21 (44.68)	
**Quantity of HBV-DNA**			0.42			0.81
0~1×10^3^	24 (48.98)	28 (57.14)		22 (51.46)	21 (53.85)	
>1×10^3^	25 (51.02)	21 (42.86)		21 (48.84)	18 (46.15)	
**AFP expression (ng/ml)**			0.92			0.97
≥400	22 (40.00)	25 (43.10)		18 (38.30)	19 (40.43)	
20~399	16 (29.09)	15 (25.86)		13 (27.66)	13 (27.66)	
<20	17 (30.91)	18 (31.04)		16 (34.04)	15 (31.91)	
**Gross classification of primary liver cancer**			0.45			0.52
Giant	17 (39.91)	16 (27.59)		16 (34.04)	13 (27.66)	
Massive	19 (34.55)	15 (25.86)		14 (29.79)	11 (23.40)	
Nodular	15 (27.27)	24 (41.38)		14 (29.79)	21 (44.68)	
Diffuse	4 (7.27)	3 (5.17)		3 (6.38)	2 (4.26)	
**Classification of PVTT**			0.57			0.65
Type I	6 (10.91)	7 (12.07)		4 (8.51)	5 (10.64)	
Type II	26 (47.27)	20 (34.48)		21 (44.68)	15 (31.91)	
Type III	19 (34.55)	25 (43.10)		18 (38.30)	22 (46.81)	
Type IV	4 (7.27)	6 (10.35)		4 (8.51)	5 (10.64)	
**Tumour metastasis**			0.72			0.90
Intrahepatic	36 (65.45)	42 (72.41)		29 (61.70)	33 (70.21)	
Lung	12 (21.82)	8 (13.79)		11 (23.40)	8 (17.02)	
Lymphonodi coeliaci	18 (32.73)	12 (20.69)		13 (27.66)	11 (23.40)	
Bone	2 (3.64)	1 (1.72)		2 (4.26)	1 (2.13)	
Kidney	1 (1.82)	1 (1.72)		1 (2.13)	1 (2.13)	
Spleen	1 (1.82)	1 (1.72)		1 (2.13)	1 (2.13)	
Omentum	0 (0.00)	1 (1.72)		0 (0.00)	1 (2.13)	
**ICIs**			0.95			> 0.99
PD-L1 inhibitor	3 (5.45)	3 (5.17)		2 (4.26)	2 (4.26)	
PD-1 inhibitor	52 (94.55)	55 (94.83)		45 (95.74)	45 (95.74)	

PVTT radiotherapy group, Radiotherapy+ICIs+Bevacizumab; Control group, ICIs+Bevacizumab; ICIs, immuno-checkpoint inhibitors; CNCL, China Liver Cancer Staging; ECOG, Eastern Cooperative Oncology Group; NAFLD, non-alcoholic fatty liver disease; APF, alpha-fetoprotein; PVTT, portal vein tumour thrombosis; PD-L1, programmed cell death-ligand1; PD-1, programmed cell death-1.

### 3.2 Analysis of indicators before and after radiotherapy of PVTT in patients with HCC

A total of 55 patients with PVTT radiotherapy and ICIs plus bevacizumab therapy group, the hemoglobin, neutrophils, lymphocytes, CRP, albumin, total bilirubin, ALT, AST, prothrombin time, CD4, CD8 in routine analysis of blood, liver function and coagulation were collected within 1 week before and 2 weeks after PVTT radiotherapy, NLR and CD4/CD8 lymphocyte ratio were calculated (**Table 2**). All the indexes mentioned above were tested by paired t-test, and the immune-related indexes including neutrophil (3.09 ± 1.39 *vs.* 4.97 ± 1.65, *t* = 12.68, *P <* 0.05), lymphocyte (0.96 ± 0.43 *vs.* 0.45 ± 0.27, *t* = 8.27, *P <* 0.05), CRP (25.30 ± 38.35 *vs.* 41.87 ± 41.88, *t* = 3.18, *P <* 0.05), CD4 (490.33 ± 54.57 *vs.* 295.96 ± 35.26, *t* = 45.34, *P <* 0.05) and CD8 (270.93 ± 31.24 *vs.* 186.47 ± 24.30, *t* = 8.27, *P <* 0.05) before and after PVTT radiotherapy were statistically significant (*P <* 0.05). NLR increased from 3.57 ± 1.73 to 14.98 ± 10.74 (*t* = 8.24, *P <* 0.05), CD4/CD8 ratio decreased from 1.81 ± 0.10 to 1.59 ± 0.11 (*t* = 17.23, *P* < 0.05). Shown in **Table 2**.

**Table 2:** Comparison of the index before and after radiotherapy for PVTT in the PVTT radiotherapy group (Radiotherapy + ICIs + Antiangiogenic) (n=55).

	Before radiotherapy (x±s)	After radiotherapy (x±s)	*t*	*P*
Hemoglobin(g/L)	129.69 ± 20.78	127.78 ± 20.90	0.96	0.34
Neutrophil(10^9^/L)	3.09 ± 1.39	4.97 ± 1.65	12.68	< 0.05
Lymphocyte(10^9^/L)	0.96 ± 0.43	0.45 ± 0.27	8.27	< 0.05
NLR	3.57 ± 1.73	14.98 ± 10.74	8.24	< 0.05
CRP (mg/L)	25.30 ± 38.35	41.87 ± 41.88	3.18	< 0.05
Albumin (g/dL)	38.85 ± 4.54	44.28 ± 45.78	0.89	0.38
TBIL (μmol/L)	18.35 ± 12.77	25.83 ± 59.43	1.10	0.28
ALT (U/L)	52.55 ± 46.98	40.27 ± 24.27	1.80	0.07
AST (U/L)	65.27 ± 48.75	56.13 ± 38.65	1.30	0.20
PT (s)	14.00 ± 1.23	13.88 ± 1.29	0.98	0.33
CD4(a/uL)	490.33 ± 54.57	295.96 ± 35.26	45.34	< 0.05
CD8(a/uL)	270.93 ± 31.24	186.47 ± 24.30	31.43	< 0.05
CD4/CD8 ratio	1.81 ± 0.10	1.59 ± 0.11	17.23	< 0.05

PVTT, portal vein tumour thrombosis; ICIs, immuno-checkpoint inhibitors; NLR, Neutrophil-to-Lymphocyte Ratio; CRP, C-reactive protein; TBIL, Total bilirubin; ALT, Alamine aminotransferase; AST, Aspartate aminotransferase; PT, Prothrombin time.

### 3.3 Survival analysis (PFS, ORR, and DCR)

**Figure 1:**
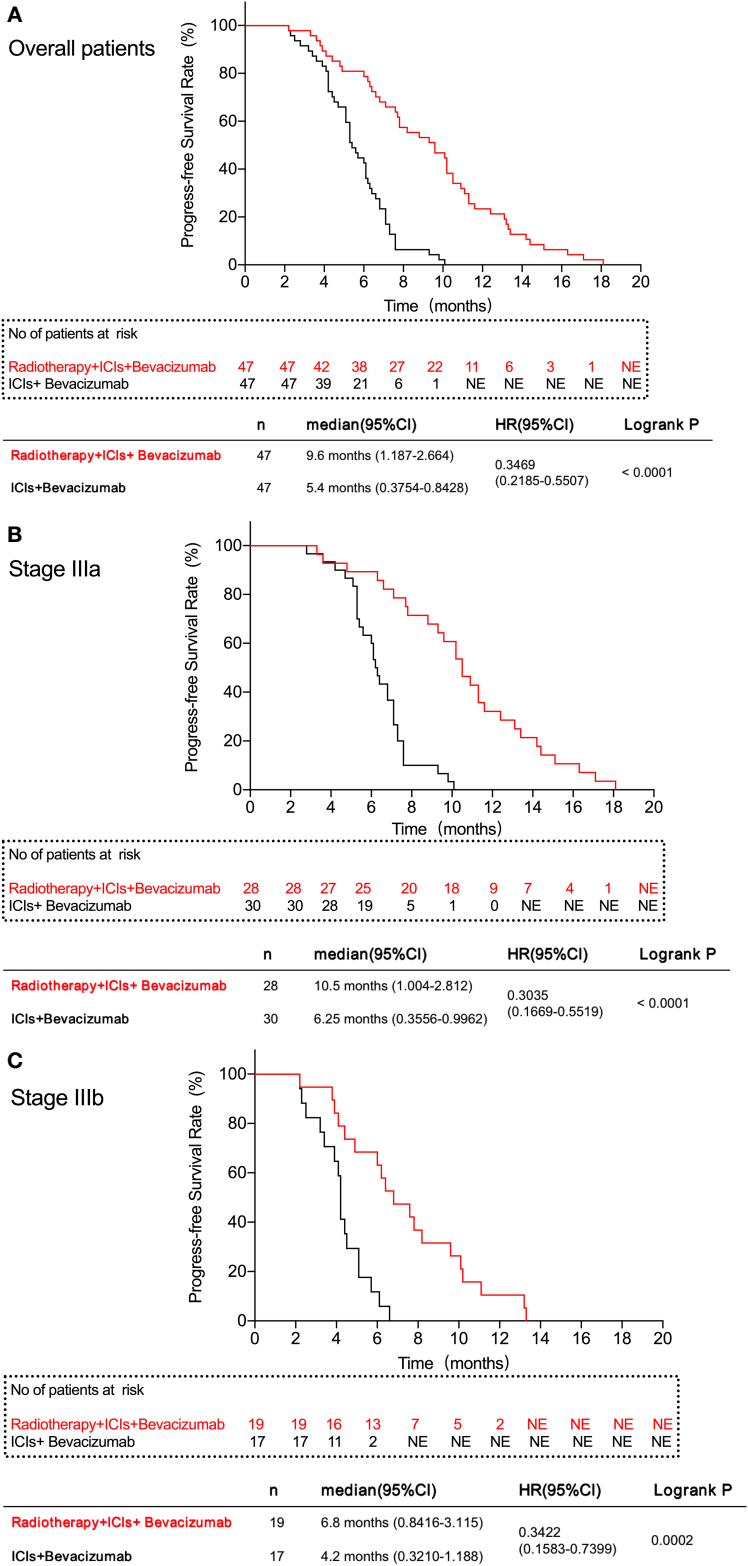
Progress-free survival rate of patients in this study. **(A)** All patients in the two groups. **(B)** patients with IIIa stage in the two groups. **(C)** patients with IIIb stage in the two groups. The results show that the PVTT radiotherapy group had a better FPS rate than the control group.

The median PFS of patients in PVTT radiotherapy group was 9.6 months, and the PFS rates at 3, 6, 9, and 12 months were 46 (97.87%), 38 (80.85%), 25 (53.19%), and 11 (23.40%), respectively. The median survival PFS was 5.4 months in the control group, and the PFS rates at 3, 6, 9, and 12 months were 43 (91.49%), 21 (44.68%), 3 (6.38%), and 0 (0.00%), respectively (**Table 3**). PFS rates (**Figure 1A**) and stage IIIa (**Figure 1B**) and IIIb (**Figure 1C**) PFS rates of the PVTT radiotherapy group were better than those of the control group (*P* < 0.001), as shown in **Figure 1**.

**Table 3:** Survival analysis and response evaluation of patients in the two groups after PSM (1:1) (RECIST 1.1 version).

	PVTT radiotherapy group (Radiotherapy+ICIs+ Bevacizumab)			Control group (ICIs+Bevacizumab)			*P*
	Total (n=47)	IIIa stage (n=28)	IIIb stage (n=19)	Total (n=47)	IIIa stage (n=30)	IIIb stage (n=17)	
**mPFS** [months (95% CI)]	9.6 (1.187-2.664)	10.5 (1.004-2.812)	6.8 (0.8416-3.115)	5.4 (0.3754-0.8428)	6.25 (0.3556-0.9962)	4.2 (0.3210-1.188)	*< 0.001*
**PFS Rate [n (%)]**							*< 0.001*
3 Months	46 (97.87)	28 (100.00)	18 (94.74)	43 (91.49)	29 (96.67)	14 (82.35)	
6 Months	38 (80.85)	25 (89.29)	13 (68.42)	21 (44.68)	19 (63.33)	2 (11.76)	
9 Months	25 (53.19)	19 (67.86)	6 (31.58)	3 (6.38)	3 (10.00)	0 (0.00)	
12 Months	11 (23.40)	9 (32.14)	2 (10.53)	0 (0.00)	0 (0.00)	0 (0.00)	
**Best response [n (%)]**							*0.0351*
CR	5 (10.64)	3 (10.71)	2 (10.53)	2 (4.26)	1 (3.33)	1 (5.88)	
PR	18 (38.30)	12 (42.86)	6 (31.58)	11 (23.40)	9 (30.00)	2 (11.76)	
SD	23 (48.94)	13 (46.43)	10 (52.63)	26 (55.32)	18 (60.00)	8 (47.06)	
PD	1 (2.13)	0 (0.00)	1 (5.26)	8 (17.02)	2 (6.67)	6 (35.29)	
**ORR [n (%)]**	23 (48.94)	15 (53.57)	8 (42.11)	13 (27.66)	10 (33.33)	3 (17.65)	*0.0339*
**DCR [n (%)]**	46 (97.87)	28 (100.00)	18 (94.74)	39 (82.98)	28 (93.33)	11 (64.71)	*0.0141*

PSM, propensity score matching; RECIST, Response Evaluation Criteria in Solid Tumors; ICIs, immuno-checkpoint inhibitors; mPFS, Median progression-free survival; CR, complete response; PR, partial response; SD, stable disease; PD, progression disease; ORR (objective response rate)= CR+PR; DCR (disease control rate)= CR+PR+SD. The italic values means that the data were statistically significant.

**Figure 2:**
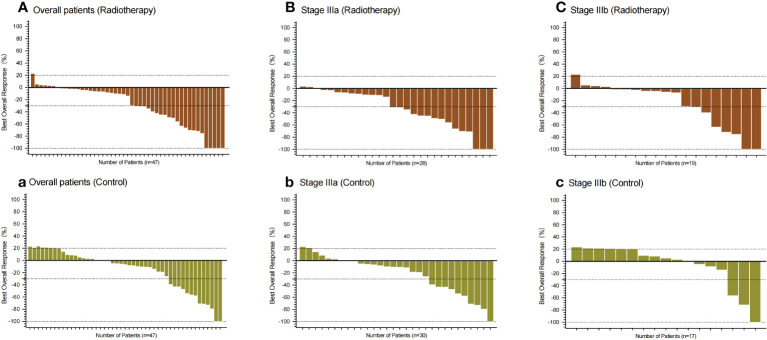
Waterfall plot showing the best percentage change from baseline in the sum of the target lesions in patients. **(A–C)** All patients, patients with IIIa stage, and patients with IIIb stage in the PVTT radiotherapy group, respectively. **(a, b, c)** All patients, patients with IIIa stage, and patients with IIIb stage in the control group, respectively. Assessed using RECIST1.1 with image measurements before and after treatment. There were no statistically significant differences between the two groups in both subgroups.

**Figure 3:**
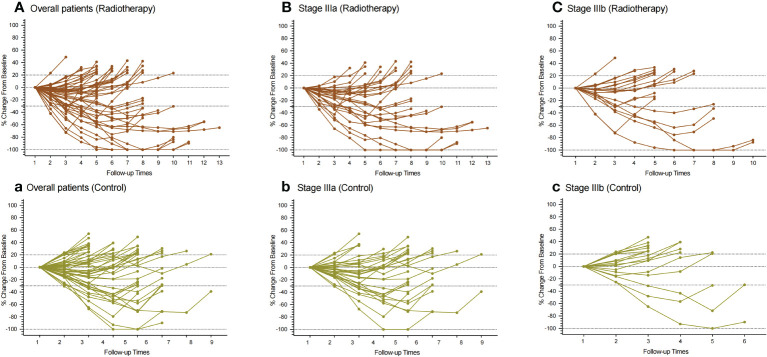
Spider plot showing the regression or growth changing from baseline in the sum of the target lesions of patients. **(A–C)**: All patients, patients with IIIa stage, and patients with IIIb stage in the PVTT radiotherapy group, respectively. **(a, b, c)** All patients, patients with IIIa stage, and patients with IIIb stage in the control group, respectively. Assessed using RECIST1.1 with image measurements before and after treatment. There were no statistically significant differences between the two groups in both subgroups.

Compared with the control group, the patients in PVTT radiotherapy group with CR were five cases *vs.* two cases (10.64% *vs.* 4.26%), PR were 18 cases *vs.* 11 cases (38.30% *vs.* 23.40%), SD patients were 23 cases *vs.* 26 cases (48.94% *vs.* 55.32%), PD patients were one case *vs.* eight cases (2.13% *vs.* 27.66%), including one patient with hyper-progression in the control group, ORR were 23 cases *vs.* 13 cases (48.94% *vs.* 27.66%), and DCR were 46 cases *vs.* 39 cases (97.87% *vs.* 82.98%). There were significant differences in the best response between the two groups (*P* = 0.0351), ORR (*P* = 0.0339) and DCR (*P* = 0.0141) (**Table 3**). Based on RECISIT1.1, the waterfall plot showed optimal tumour regression in the PVTT radiotherapy combined with systemic therapy group (**Figure 2A**) and systemic therapy-only groups (**Figure 2a**), and there were no significant differences in optimal tumour regression between the two groups at stage IIIa (**Figure 2B, b**) and IIIb (**Figure 2C, c**). The spider plot shows regression or growth in the PVTT radiotherapy combined with systemic therapy group (**Figure 3A**), and systemic therapy-only groups (**Figure 3a**) at each follow-up, and there were no significant differences between the two groups in terms of stage IIIa (**Figure 3B, b**) and IIIb (**Figure 3C, c**). A total of 42 patients experienced OS events by the end of data collection in June 2022 of this research, and the maturity of the current OS data reached 37.17%. Continued follow-up of OS data will be presented in further research.

### 3.4 Analysis of risk factors of PFS

Sex, age, smoking, alcohol consumption, diabetes, hypertension, cardiovascular disease, child-pugh grade, ECOG-PS score, whether B viral hepatitis, whether cirrhosis, AFP lever before treatment (< 400ng/ml or ≥400 ng/ml), HBV-DNA lever (0-1×10^3^ or >1×10^3^), whether giant HCC, PVTT type (I-II or III-IV), whether extrahepatic metastasis, whether PVTT was treated with radiotherapy, and whether there was reduction of PVTT were analyzed by univariate Cox analysis. The results suggested that the expression of AFP before treatment (HR 1.950, 95%*CI* 1.271-2.992, *P* = 0.002), giant HCC (HR 2.211, 95%*CI* 1.397-3.499, *P* = 0.001), PVTT type (HR 2.211, 95%*CI* 1.859-4.788, *P* < 0.001), extrahepatic metastasis (HR 1.921, 95%*CI* 1.177-3.133, *P* = 0.009), radiotherapy for PVTT (HR 0.227, 95%*CI* 0.135-0.328, *P* < 0.001), and reduction of PVTT (HR 0.107, 95%CI 0.049-0.233, *P* < 0.001) were the influencing factors of PFS (**Table 4**). The *P* value equal to 0.2 was used as the boundary to screen out the factors with significant differences in *P* < 0.2 of factors mentioned above, multivariate Cox regression analysis was conducted to further analyze the influencing factors of PFS. The results has showed that: the level of AFP before treatment (HR 1.702, 95%*CI* 1.081-2.681, *P* = 0.022), giant HCC (HR 1.753, 95%*CI* 1.064-2.889, *P* = 0.028), PVTT type (HR 1.796, 95%*CI* 1.061-3.041, *P* = 0.029), extrahepatic metastasis (HR 2.105, 95%*CI* 1.240-3.572, *P* = 0.006), radiotherapy for PVTT (HR 0.231, 95%*CI* 0.133-0.401, *P* < 0.001), and the reduction of PVTT (HR 0.175, 95%*CI* 0.073-0.416, *P* < 0.001) were the independent influencing factors for PFS (**Table 4**).

**Table 4:** Univariate and multivariate Cox regression analyses of risk factors for progression-free survival following PSM (1:1).

Variable	Univariate Cox Analysis			Multivariate Cox analysis		
	HR	95% *CI*	*P* value	HR	95% *CI*	*P* value
Sex (male *vs.* female)	1.103	0.612-1.989	0.745			
Age (years) (≤ 60 *vs*. > 60)	1.105	0.672-1.643	0.827			
Smoking (yes *vs.* no)	0.911	0.599-1.385	0.662			
Alcohol consumption (yes *vs.* no)	0.902	0.581-1.400	0.645			
Diabetes (yes *vs.* no)	0.757	0.445-1.286	0.303			
Hypertension (yes *vs.* no)	1.177	0.563-2.462	0.666			
Cardiovascular disease (yes *vs.* no)	1.641	0.401-6.720	0.491			
Child-Pugh grading (A *vs*. B)	1.460	0.804-2.649	0.214			
ECOG-PS (0 *vs*.1)	0.721	0.589-1.442	0.922			
Hepatitis B (yes vs. no)	0.979	0.530-1.808	0.945			
Liver cirrhosis (yes *vs.* no)	1.330	0.879-2.012	0.177			
HBV-DNA (0~1×10^3^ *vs.*>1×10^3^)	1.153	0.758-1.754	0.506			
AFP (<400 ng/ml *vs*. ≥ 400ng/ml)	*1.950*	*1.271-2.992*	*0.002*	*1.702*	*1.081-2.681*	*0.022*
Gross (giant *vs.* others)	*2.211*	*1.397-3.499*	*0.001*	*1.753*	*1.064-2.889*	*0.028*
Extrahepatic metastasis (yes *vs.* no)	*1.921*	*1.177-3.133*	*0.009*	*2.105*	*1.240-3.572*	*0.006*
PVTT (Type I-II *vs*.Type III-IV )	*2.984*	*1.859-4.788*	*<0.001*	*1.796*	*1.061-3.041*	*0.029*
PVTT Radiotherapy (yes vs. no)	*0.227*	*0.135-0.382*	*<0.001*	*0.231*	*0.133-0.401*	*<0.001*
Reduction of PVTT (yes vs. no)	*0.107*	*0.049-0.233*	*<0.001*	*0.175*	*0.073-0.416*	*<0.001*

ECOG-PS, Eastern Cooperative Oncology Group performance status score; APF, alpha-fetoprotein; PVTT, portal vein tumour thrombosis. The italic values means that the data were statistically significant.

### 3.5 Toxicity

There were no significant differences in AEs between the PVTT radiotherapy and control groups (*P* > 0.05). The main AEs of any grade in the two groups included weight loss (70.21% and 65.96%), hypertension (48.94% and 51.06%), decreased appetite (46.81% and 53.19%), proteinuria (46.81% and 48.94%), hand-foot syndrome (42.55% and 38.20%), fatigue (40.43% and 42.55%), hypothyroidism (29.79% and 31.91%), pruritus (29.79% and 34.04%)*, et al.* (**Table 5**). No significant increase in cTn, electrocardiographic (ECG) changes, or clinical symptoms of cardiac dysfunction were found in either group. Patients of AEs Grade ≥ 3 in PVTT radiotherapy group compared with the control group, there were 6 cases *vs.* 7 cases with weight loss (*P* = 0.765), 11 cases *vs. 9* cases with hypertension (*P* = 0.614), 3 cases *vs.* 6 cases with decreased appetite (*P* = 0.293), and 5 cases *vs.* 6 cases with proteinuria (*P* = 0.748) in both groups, 5 cases *vs.* 6 cases of hand-foot syndrome (*P* = 0.748), and 4 cases *vs.* 4 cases of pruritus (*P* = 1.000), respectively for grade ≥ 3 AEs (**Table 5**).

**Table 5:** Treatment-related adverse events in the two groups following PSM (1:1).

Adverse Event	Any Grade			Grade ≥ 3		
	PVTT radiotherapy group (n=47)	Control group (n=47)	*P* value	PVTT radiotherapy group (n=47)	Control group (n=47)	*P* value
Weight loss	33 (70.21)	31 (65.96)	0.658	6 (12.77)	7 (14.89)	0.765
Hypertension	23 (48.94)	24 (51.06)	0.837	11 (23.40)	9 (19.15)	0.614
Decreased appetite	22 (46.81)	25 (53.19)	0.536	3 (6.38)	6 (12.77)	0.293
Proteinuria	22 (46.81)	23 (48.94)	0.836	5(10.64)	6 (12.77)	0.748
Hand-foot syndrome	20 (42.55)	18 (38.20)	0.674	5 (10.64)	6 (12.77)	0.748
Fatigue	19 (40.43)	20 (42.55)	0.834	0 (0.00)	1 (2.13)	0.315
Hypothyroidism	14 (29.79)	16 (34.04)	0.658	0 (0.00)	0 (0.00)	–
Pruritus	14 (29.79)	15 (31.91)	0.823	4 (8.51)	4 (8.51)	1.000
Hypoalbuminemia	13 (27.66)	12 (25.53)	0.815	0 (0.00)	1 (2.13)	0.315
Headache	12 (25.53)	10 (21.28)	0.626	0 (0.00)	0 (0.00)	–
Rash	10 (21.28)	13 (27.66)	0.472	2 (4.26)	1 (2.13)	0.557
Increased AST	10 (21.28)	9 (19.15)	0.797	1 (2.13)	0 (0.00)	0.315
Increased ALT	9 (19.15)	11 (23.40)	0.614	1 (2.13)	0 (0.00)	0.315
Nausea	9 (19.15)	10 (21.28)	0.797	0 (0.00)	0 (0.00)	–
Anemia	8 (17.02)	7 (14.89)	0.778	0 (0.00)	1 (21.3)	0.315
Increased TBi	7 (14.89)	7 (14.89)	1.000	0 (0.00)	1 (2.13)	0.315
Arthralgia	6 (12.77)	8 (17.02)	0.562	0 (0.00)	0 (0.00)	–
Diarrhea	5 (10.64)	8(17.02)	0.370	1 (2.13)	0 (0.00)	0.315
Vomiting	5 (10.64)	6 (12.77)	0.748	0 (0.00)	0 (0.00)	–
Edema	5 (10.64)	3 (6.38)	0.460	0 (0.00)	0 (0.00)	–
Thrombocytopenia	4 (8.51)	3 (6.38)	0.694	1 (2.13)	1 (2.13)	1.000
Leukopenia	3 (6.38)	8 (17.02)	0.109	0 (0.00)	1 (2.13)	0.315
Gingival bleeding	3 (6.38)	4 (8.51.)	0.694	0 (0.00)	0 (0.00)	–
Elevated uric acid	2 (4.26)	2 (4.26)	1.000	0 (0.00)	0 (0.00)	–
Neutropenia	2 (4.26)	6 (12.77)	0.139	0 (0.00)	1 (2.13)	0.315
Dysphonia	2 (4.26)	1 (2.13)	0.557	0 (0.00)	0 (0.00)	–
Hyperglycemia	1 (2.13)	2 (4.26)	0.557	0 (0.00)	0 (0.00)	–
Pneumonitis	1 (2.13)	0 (0.00)	0.315	0 (0.00)	0 (0.00)	–

PVTT radiotherapy group: Radiotherapy+ICIs+ Bevacizumab; Control group: ICIs+ Bevacizumab. Data were presented as n (%). ICIs, immuno-checkpoint inhibitors; ALT, alanine aminotransferase; AST, Aspartate aminotransferase; TBi, total bilirubin.

## 4 Discussion

The systemic therapy progress of advanced HCC is slow. As sorafenib became the first approved system treatment in 2007, breakthroughs in HCC treatment over the next 10 years have been rare and long-term drugs are lacking. The efficiency of sorafenib is low and limited to improving survival. Additionally, notable adverse effects indicated the need to acquire more effective therapies with lower toxicity against advanced HCC. In recent years, ICIs have become a hot area of clinical research in advanced HCC. Checkmate040 (4) (phase I/II) is a landmark study in the history of HCC immunotherapy, and its results have established nivolumab as a second-line therapy for advanced HCC. The Checkmate-459 (11) study enrolled patients with advanced HCC who were ineligible for surgery or local treatment and patients who progressed after surgery or local treatment. The results have shown clinically meaningful improvements in OS, ORR, and CR rates, but they did not meet the primary endpoint of OS. This study suggests that although monotherapy with ICIs has improved OS and ORR compared to sorafenib, it does not have absolute advantages, and ICIs combined therapy with other methods may be a better choice. Several studies conducted in the last 5 years have reported that ICIs combined with anti-angiogenic therapy have a good effect and can further improve the survival rate of patients. GO30140 (12) and Imbrave150 (8) showed that atezolizumab combined with bevacizumab as the first-line treatment in patients with advanced HCC can improve the ORR and significantly prolong the OS of patients to 17.1 and 19.2 months, respectively. The subgroup data of 194 Chinese patients in the Imbrave150 study showed that the median OS was 24 months (13), which has advanced past the bottleneck of HCC treatment in the past decade. The studies mentioned above indicate that ICIs are feasible and safe for the treatment of advanced HCC; however, these results also showed that the ORR of single-agent ICIs was low, and the combination treatment of ICIs with other methods, such as anti-angiogenic therapy, is promising for future HCC treatment.

Radiotherapy can change the microenvironment of tumour cells, promote the production of T cells and immune infiltration, and stimulate the body to produce anti-tumour immune effects. Radiotherapy can induce immunogenicity of death in tumour cells, release inflammatory factors and cytokines, and generate new tumour antigens. Antigen-presenting cells (APCs) can enter the tumour cells and access the tumour antigen, causing a systemic anti-tumour effect mediated by the immune system, resulting in “remote effects” (14). Abulimiti et al. confirmed that radiotherapy combined with sorafenib improved the survival of patients with HCC, with a median OS of 11.4 months and a median PFS of 6 months (15). Furthermore, another study (16) indicated that the mPFS of patients with advanced HCC treated with IMRT combined with apatinib was 7.8 months and the ORR was 15%. All the studies mentioned above indicate that radiotherapy has a synergistic effect on systemic anti-tumour therapy. Anti-angiogenic therapy can normalise the blood vessels of tumours and enhance the infiltration of T cells simultaneously (17), providing a theoretical basis for radiotherapy combined with immunotherapy and anti-angiogenic therapy. HCC is a typical inflammation-related tumour (18), and its microenvironment is primarily composed of cellular components, such as tumour-associated macrophages, tumour-associated neutrophils, tumour-infiltrating lymphocytes, tumour-associated fibroblasts, non-cellular components, and extracellular stromal cytokines. The immune-related microenvironment plays an important role in HCC progression, immune escape, and treatment resistance. As an evaluation index of the systemic inflammatory response, the NLR is an independent prognostic factor for various malignant tumours, such as gastric, lung, and colorectal cancers, and studies have also confirmed that NLR can be used as an indicator to evaluate the prognosis of patients with HCC (19). Our study showed that the NLR of the peripheral venous blood increased after radiotherapy in the PVTT radiotherapy group, reflecting an obvious inflammatory reaction in the body after radiotherapy. Moreover, although the CD4 and CD8 counts decreased to a certain degree after radiotherapy for PVTT, the CD4-to-CD8 ratio showed a statistically significant decrease, indicating an increase in the proportion of cytotoxic T cells with killing function and the enhancement of body immunity. ICIs and anti-angiogenic therapies are theoretically feasible based on inflammatory reactions and immune enhancement (20).

Tumour antigen release is a key link in immunotherapy, and therapies that can increase tumour neoantigens should enhance the effects of immunotherapy (11). In our study, radiotherapy with PVTT caused necrosis of the tumour tissue, and the exposure to tumour antigens promoted the inflammatory response of the body, which changed some immune-related indicators of the body, thereby improving the efficacy of ICIs treatment. The median PFS of the PVTT radiotherapy group was 4.2 months longer than that of the control group; the ORR was 48.94% *vs*. 27.66%, and the DCR was 97.87% *vs*. 82.98%, indicating the advantages of radiotherapy for PVTT in the treatment of HCC. Subgroup analysis suggested that the therapeutic effect was directly related to staging. The IMbrave150 study indicated that the main factors affecting the long-term survival of patients with PFS and OS after treatment with atezolizumab plus bevacizumab included viral infection and AFP levels. In this retrospective study, multivariate Cox analysis showed that AFP expression, PVTT type, liver tumour size, and PVTT radiotherapy were independent prognostic factors affecting PFS. Our retrospective study also indicated that the highest incidences of AEs were weight loss, hypertension, and decreased appetite, most of which were grade 1-2. Common immune-related AEs (irAEs) were pruritus, rash, and hypothyroidism, and no serious irAEs were observed in patients in either group.

## 5 Conclusion

Radiotherapy for PVTT in HCC can quickly eliminate the tumour tissue and induce large quantities of neoplastic *de novo* antigens which activate immunity for the immunotherapy response. Combined ICIs and anti-angiogenic therapy after radiotherapy for PVTT can improve survival and is well-tolerated. Data from prospective clinical studies with higher levels of evidence are required to guide clinical applications, and relevant clinical studies should be conducted in the future.

## Data availability statement

The raw data supporting the conclusions of this article will be made available by the authors, without undue reservation.

## Ethics statement

The studies involving humans were approved by The Second Affiliated Hospital of Chongqing Medical University Research Ethics Committee. The studies were conducted in accordance with the local legislation and institutional requirements. The human samples used in this study were acquired from primarily isolated as part of your previous study for which ethical approval was obtained. Written informed consent for participation was not required from the participants or the participants’ legal guardians/next of kin in accordance with the national legislation and institutional requirements.

## Author contributions

CT: Conceptualization, Data curation, Formal Analysis, Investigation, Methodology, Software, Writing – original draft, Writing – review & editing. QH: Data curation, Methodology, Writing – review & editing. JF: Data curation, Methodology, Writing – review & editing. ZL: Data curation, Methodology, Writing – review & editing. YP: Data curation, Methodology, Writing – review & editing. JG: Formal Analysis, Funding acquisition, Project administration, Resources, Supervision, Validation, Visualization, Writing – review & editing.
